# Diosgenin restores Aβ-induced axonal degeneration by reducing the expression of heat shock cognate 70 (HSC70)

**DOI:** 10.1038/s41598-018-30102-8

**Published:** 2018-08-03

**Authors:** Ximeng Yang, Chihiro Tohda

**Affiliations:** Division of Neuromedical Science, Department of Bioscience, Institute of Natural Medicine, University of Toyama, Toyama, Japan

## Abstract

We previously found diosgenin, an herbal drug-derived steroid sapogenin, to be remarkably effective at restoring Aβ-induced axonal degeneration and improving memory function in model of Alzheimer’s disease (AD), 5XFAD mouse. In this study, we investigated the downstream signaling of diosgenin and explored new therapeutic targets in AD. We showed that the expression of heat shock cognate (HSC) 70 was increased in Aβ-treated neurons and in 5XFAD mice but was decreased by diosgenin treatment. In addition, knockdown of HSC70 significantly promoted axonal growth in neurons. As an association molecule of HSC70 in neurons, α-tubulin was detected by immunoprecipitation. After Aβ treatment, α-tubulin expression was greatly reduced in the degenerated axons, suggesting that a decline in α-tubulin may be one of the factors which correlates with axonal disruption in AD pathology. We hypothesized that the degradation of α-tubulin is triggered by the chaperone activity of HSC70. However, diosgenin significantly normalized the α-tubulin level, a potentially critical process for axonal formation. Our study indicated that reducing the HSC70 level is a new possible therapeutic target of axonal regeneration in AD.

## Introduction

Alzheimer’s disease (AD) is a progressive neurodegenerative disorder, characterized by deposition of amyloid β (Aβ) in the brain and remains a refractory disease. Although decades of research have focused on several agents that reduce the Aβ level, these agents have failed in phase 2 or 3 clinical trials^1^ suggesting that regaining memory function in spite of a decline in Aβ in the brain is difficult. We have hypothesized that damaged neural networks must be repaired to recover the memory deficits in AD. Therefore, we have explored new anti-AD drug candidates to restore axonal degeneration and synapse formation, which directly relate to memory recovery.

We previously found that diosgenin, an herbal drug-derived steroid sapogenin, restored Aβ-induced axonal atrophy in neurons and improved memory function in a mouse model of AD, 5XFAD mice^2^. Furthermore, diosgenin administration significantly reduced the presence of degenerated axons associated with Aβ plaques in the 5XFAD mouse brain. The 5XFAD (Tg6799) mouse co-expresses mutant human amyloid precursor protein (APP; the Swedish mutations: K670N and M671L; the Florida mutation: I716V; the London mutation: V717I) and presenilin-1 (PS1; M146L and L286V) genes specifically in neurons^3^. These five familial AD mutations act together to increase the level of Aβ_1–42_ peptide in the brain. Deposition of Aβ plaques begins at 2 months old, and memory dysfunction is observed beginning at 4 months old in 5XFAD mice. Diosgenin is a constituent of Dioscorea rhizome and other crude drugs and has several pharmacological activities, such as anti-cancer^4^, anti-diabetic neuropathy^5^, anti-cognitive deficit^6^ and anti-food allergy^7^ effects. Previously, we have shown that a cell surface receptor for diosgenin, 1,25D_3_-MARRS (membrane-associated rapid response steroid-binding receptor) leads to axonal regrowth^2^ and memory enhancement^8^.

In this study, we aimed to investigate the downstream signaling elicited by diosgenin as it directly relates to axonal regrowth in AD. The goal of this study was not only to provide scientific support for the use of diosgenin as a curative drug for AD but also to suggest new possible therapeutic targets in AD by exploring diosgenin signaling.

## Results

### Exploring the effects of diosgenin on protein expression in the 5XFAD mouse cortex

We have previously found that diosgenin administration significantly recovers memory deficits in the 5XFAD mice. In addition, pNF-H-positive abnormal bulb-like swollen axons located within the amyloid plaques were significantly reduced in the 5XFAD brains by diosgenin administration^2^. Therefore, we hypothesized that diosgenin administration would result in dynamic changes leading to the recovery of axonal degeneration and memory deficits. First of all, we identified proteins whose expression levels were altered by diosgenin administration in the 5XFAD mouse brain in this study.

**Figure 1:**
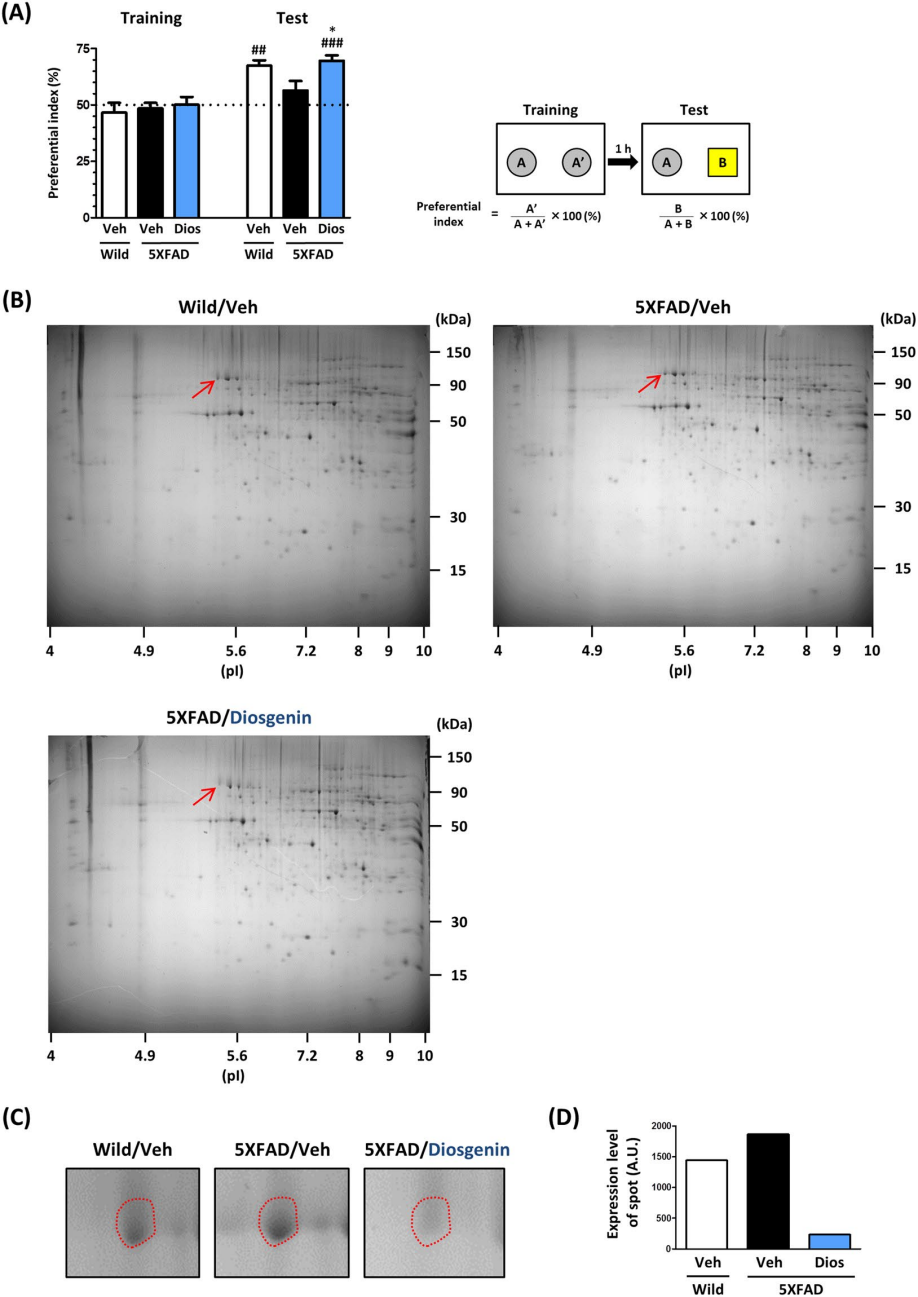
HSC70 expression in 5XFAD mouse cortex was drastically reduced by diosgenin administration. Diosgenin (0.1 µmol/kg/day, p.o.) or vehicle solution was administered to wild-type and 5XFAD mice (male, 5–6 months old) for 15 days. (**A**) On the last day of administration, an object recognition memory test was performed. The preferential indexes of the training and test session are shown. *p < 0.05 vs 5XFAD/Veh, two-tailed one-way ANOVA *post hoc* Dunnett’s test, ^##^p < 0.01, ^###^p < 0.001 vs 50%, two-tailed one sample *t*-test, n = 4 mice. (**B**) Cortex lysates were prepared, and the protein expression levels were compared on 2D-PAGE for each mouse. (**C**) Magnified images of the sections indicated by the red arrows in (**B**). This protein was more highly expressed in 5XFAD mice than in wild-type mice, but its expression was drastically decreased by diosgenin administration. This spot was cut out for MS analysis. (**D**) Quantitative value of the expression levels of the spot.

Either diosgenin (0.1 µmol/kg/day) or vehicle solution was orally administered to 5XFAD mice for 15 days. On the last day of administration, an object recognition memory test was performed. In this test, all of the mice showed equal exploratory behavior toward two objects in the training session (preferential indexes were approximately 50% in each group). In the test session, the diosgenin-administered 5XFAD mice showed a significantly higher preferential index to the new subject than the vehicle-administered 5XFAD mice (Fig. 1A), indicating that diosgenin treatment recovered the object recognition memory in 5XFAD mice. After the behavioral test, cortex lysates in each group were prepared to perform 2D-PAGE analysis (Fig. 1B). In total, 925 protein spots in wild-type, 974 spots in vehicle-treated 5XFAD and 962 spots in diosgenin-treated 5XFAD mouse were detected. Among them, the five proteins whose expression was drastically altered by diosgenin administration were identified by using MALDI-TOF/MS analysis. Heat shock cognate 70 (HSC70; protein sequence coverage: 39%, score: 309) was identified as a protein whose expression was increased in the 5XFAD mouse, but then remarkably decreased by diosgenin administration (Fig. 1C,D, respectively).

HSC70 is a molecular chaperone that belongs to the heat shock protein family^9^. HSC70 expression has been reported to be higher in AD model mice and in AD patient brains^10^. However, the direct relationship between HSC70 and axons or memory has not been clarified. Therefore, we examined whether the diosgenin-induced reduction in HSC70 is one of the beneficial events leading to axonal regrowth and memory recovery.

### Expression of HSC70 was decreased by diosgenin treatment in neurons and in the 5XFAD mouse cortex

**Figure 2:**
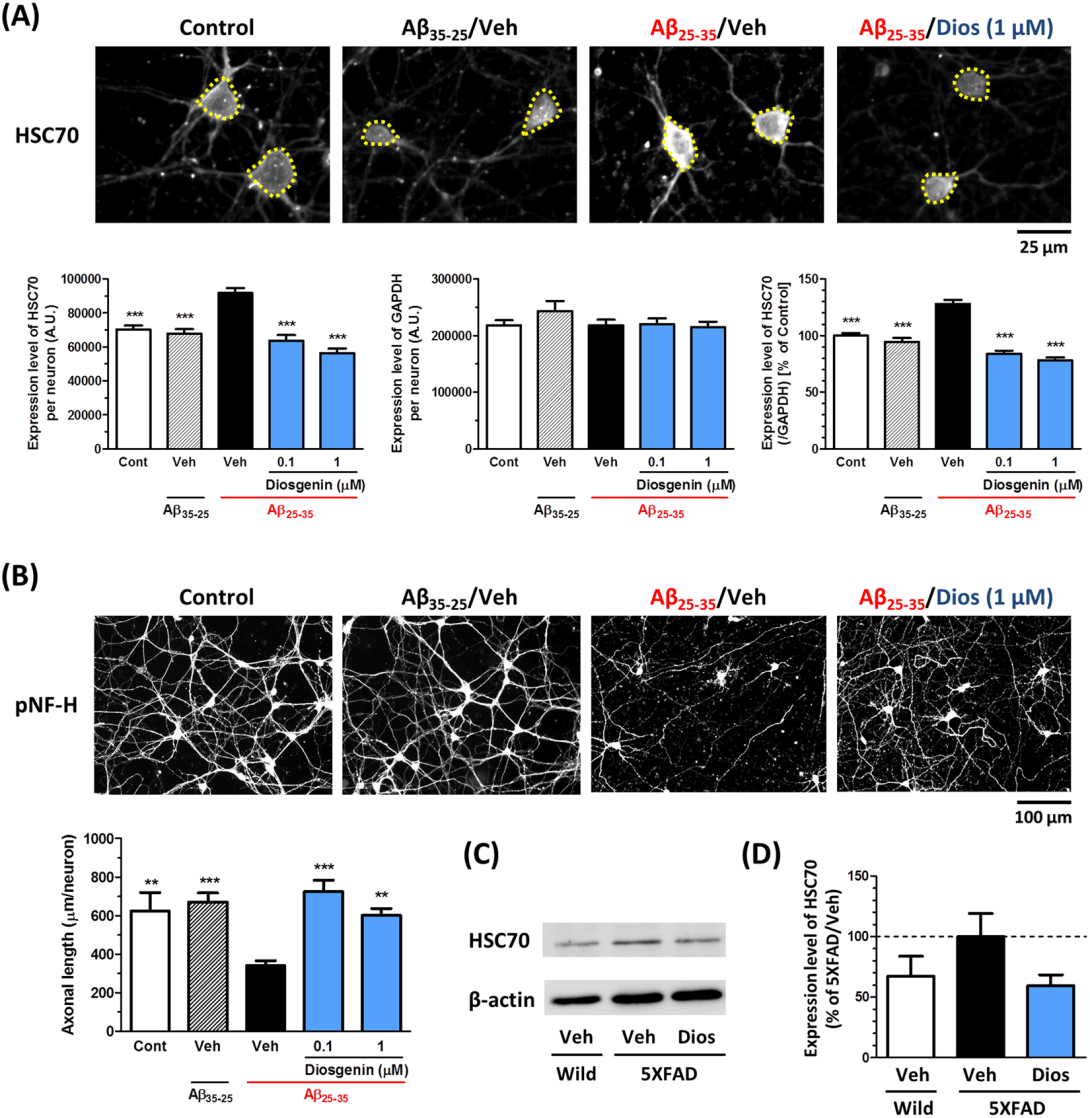
Diosgenin treatment decreased expression of HSC70 in cultured neurons and in 5XFAD mouse brains. (**A**,**B**) Mouse cortical neurons (ddY, E14) were cultured for three days and then treated with Aβ_35–25_ (10 µM) or Aβ_25–35_ (10 µM) for three days. Then, neurons were treated with diosgenin (0.1 or 1 µM) or vehicle solution (0.1% EtOH) for four days. Neurons were fixed and immunostained for HSC70 and GAPDH, or pNF-H. (**A**) The expression level of HSC70 (yellow dotted line) and GAPDH in neurons were quantified, and the expression level of HSC70 (ratio to GAPDH) were quantified in each neuron. (**B**) pNF-H-positive axonal lengths were quantified for each treatment group. **p < 0.01, ***p < 0.001, two-tailed one-way ANOVA *post hoc* Dunnett’s test. (**A**) n = 115–217 neurons, (**B**) n = 11–15 photos were quantified for these analyses. (**C**,**D**) Wild-type and 5XFAD mice (Female, 7–8 months old) were treated with diosgenin (0.1 µmol/kg/day, p.o.) or vehicle solution (sesame oil) for 18 days. (**C**) WB for HSC70 in the wild-type mouse and diosgenin- or vehicle-administered 5XFAD mouse cortex. Representative cropped images are shown for each group. The full-length blots are presented in Supplementary Fig. 2D. (**D**) Quantitative value for the expression levels of HSC70 (ratio to β-actin), n = 3–4 mice.

To confirm the results of the 2D-PAGE analysis, expression of HSC70 in neurons and in the 5XFAD brains was investigated. Treatment with Aβ_25–35_ (10 µM), but not with a negative control peptide Aβ_35–25_ (10 µM), increased the expression of HSC70 in neurons. However, post treatment with diosgenin (0.1 or 1 µM) significantly decreased its expression. On the other hand, the expression of GAPDH in neurons was not changed among all the groups (Fig. 2A). We also observed that diosgenin reduced the expression level of HSC70 in neuron lysates using WB analysis (Supplementary Fig. 1). Similar to the full-length of Aβ peptide, one of the functional domains of Aβ fragment, Aβ_25–35_, is also present in the brains of AD patients, and has a high neurotoxicity^11^. Furthermore, we previously found that both of Aβ_1–42_ and Aβ_25–35_ have similar effects on axonal atrophy in neurons^12,13^. In this study, the density of pNF-H-positive axons was also reduced by Aβ_25–35_ treatment, and its negative control peptide Aβ_35–25_ has no effect on axonal atrophy. However, diosgenin significantly restored this Aβ_25–35_ -induced axonal atrophy (Fig. 2B).

Diosgenin (0.1 µmol/kg/day) or vehicle solution was orally administered to 5XFAD mice for 18 days. On administration day 14, an object recognition memory test was performed. Vehicle-administered 5XFAD mice showed severe memory deficits, but diosgenin significantly recovered the object recognition memory in 5XFAD mice (Supplementary Fig. 2B). In the locomotion test, no significant differences in velocity were detected among the groups (Supplementary Fig. 2C). WB analysis of the cortex samples from these mice showed that HSC70 expression was higher in 5XFAD mice than in wild-type mice, but diosgenin administration decreased its expression in the 5XFAD mouse cortex (Fig. 2C,D, respectively. The full-length blots are presented in Supplementary Fig. 2D). These data suggested that diosgenin decreases HSC70 expression in neurons and in the 5XFAD mouse brain.

### Reduction in HSC70 was mediated by 1,25D_3_-MARRS signaling

We have previously identified 1,25D_3_-MARRS as a direct binding protein of diosgenin and demonstrated that inhibiting 1,25D_3_-MARRS signaling with a 1,25D_3_-MARRS neutralizing antibody or transfection of siRNA targeting 1,25D_3_-MARRS could almost completely block the diosgenin-induced axonal regrowth^2^ and the memory enhancement^8^. To clarify that one of the important factors in diosgenin signaling is the reduction in HSC70, we investigated whether the reduction in HSC70 was mediated by 1,25D_3_-MARRS signaling.

**Figure 3:**
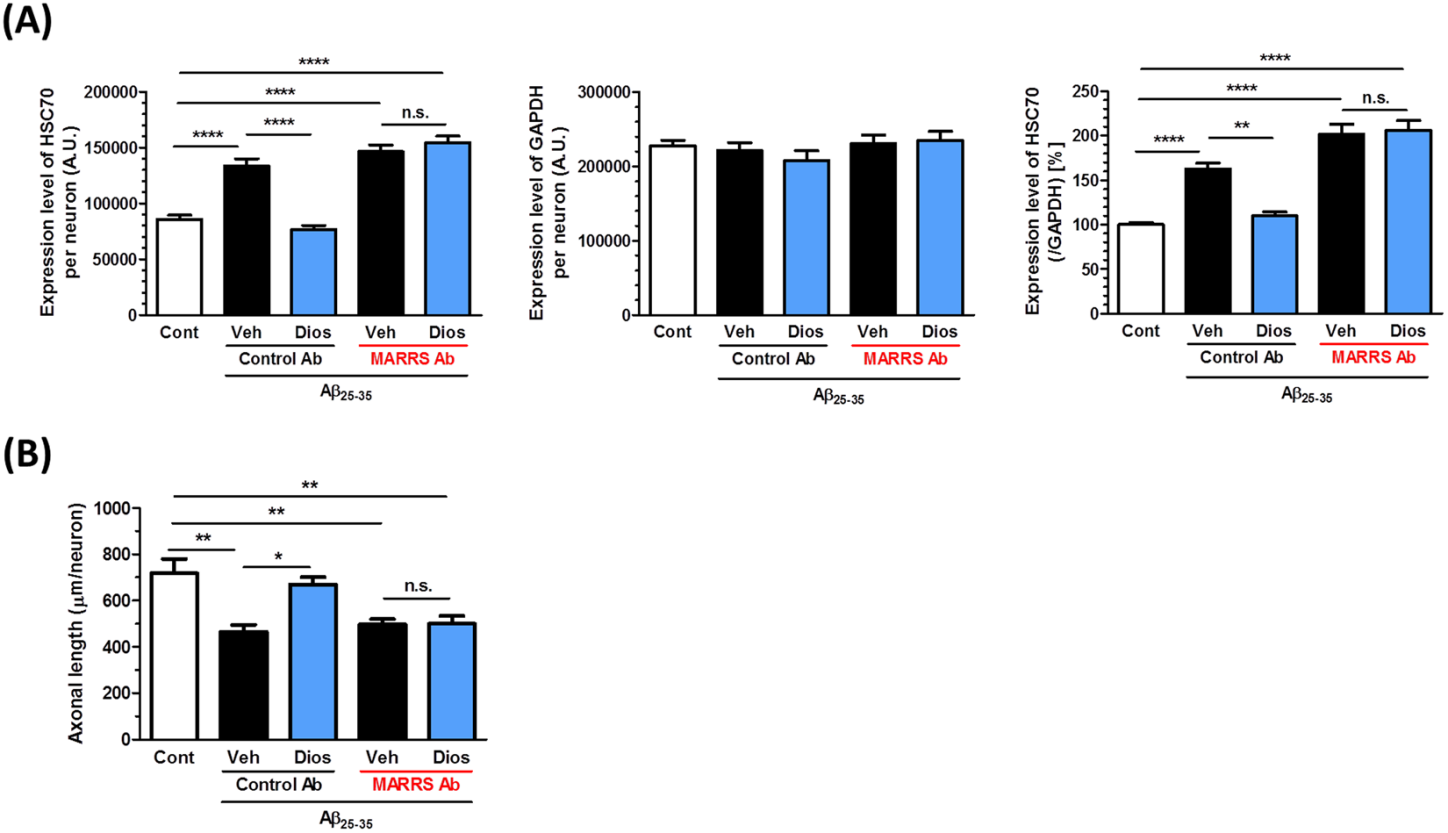
Diosgenin-induced the reduction of HSC70 and axonal regrowth are mediated by 1,25D_3_-MARRS. Mouse cortical neurons were cultured for three days and then treated with or without Aβ_25–35_ (10 µM) for three days. Three days after Aβ_25–35_ treatment, neurons were incubated with the 1,25D_3_-MARRS antibody (MARRS Ab) or normal rabbit IgG (Control Ab) for 15 minutes, followed by treatment with diosgenin (1 µM) or vehicle solution (0.1% EtOH). Four days after the treatments, the neurons were fixed and immunostained for HSC70 and GAPDH, or pNF-H. (**A**) The expression level of HSC70 and GAPDH in neurons were quantified, and the expression level of HSC70 (ratio to GAPDH) were quantified in each neuron. (**B**) pNF-H-positive axonal lengths were quantified for each treatment group. *p < 0.05, **p < 0.01, ****p < 0.0001, two-tailed one-way ANOVA *post hoc* Bonferroni’s multiple comparison test. (**A**) n = 78–243 neurons, (**B**) n = 9–14 photos were quantified for these analyses.

Three days after treatment with Aβ_25–35_ (10 µM), the neurons were treated with the 1,25D_3_-MARRS neutralizing antibody, followed by the addition of diosgenin (1 µM) 15 min later. The expression levels of HSC70 and GAPDH in neurons were quantified, and the ratio of HSC70 to GAPDH in each neuron was calculated. The diosgenin-induced reduction in HSC70 and axonal regrowth were diminished by 1,25D_3_-MARRS neutralizing antibody treatment (Fig. 3A,B, respectively), suggesting that the diosgenin-induced reduction in HSC70 and axonal regrowth are mediated by 1,25D_3_-MARRS signaling.

### Knockdown of HSC70 enhanced axonal growth in neurons

To investigate the effect of the reduction in HSC70 expression on axonal growth, an HSC70 knockdown experiment was performed using siRNA transfection. To distinguish the siRNA-transfected neurons, GFP vectors were mixed together with siRNAs, and the expression of HSC70 and axonal length were measured only in siRNA-transfected neurons.

**Figure 4:**
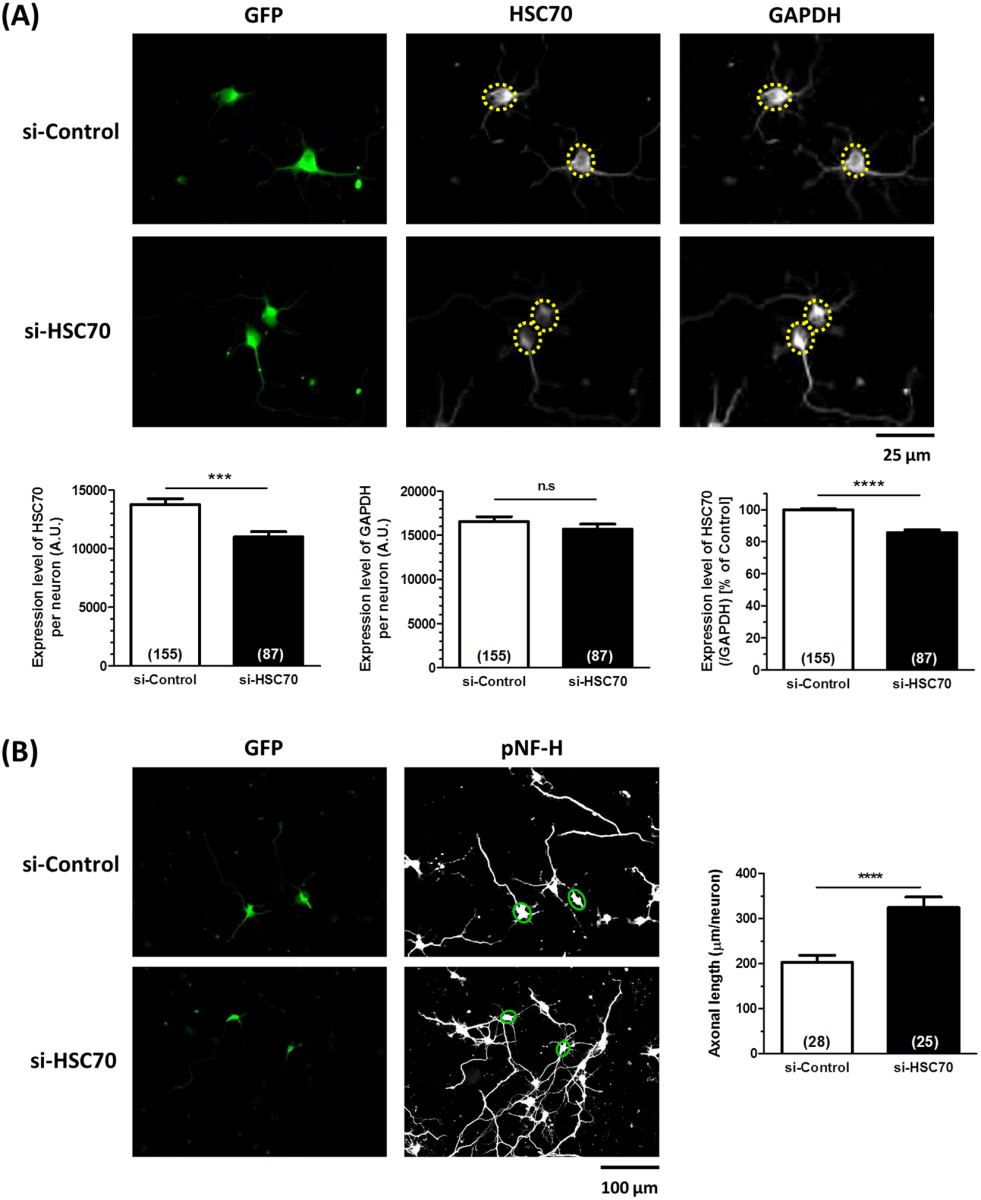
Effect of HSC70 knockdown on axonal growth. siRNA for HSC70 (300 µM) or control siRNA (300 µM) was transfected together with GFP vector into mouse cortical neurons. Four days later, neurons were immunostained with HSC70 and GAPDH, or pNF-H. (**A**) The expression level of HSC70 and GAPDH in neurons were quantified, and the expression level of HSC70 (ratio to GAPDH) were quantified in each neuron. (**B**) pNF-H-positive axonal lengths were quantified for each treatment group. ***p < 0.001, ****p < 0.0001, two-tailed unpaired *t*-test. **(A)** n = 87–155 neurons, (**B**) n = 25–28 photos were quantified for these analyses.

Four days after transfection with siRNA targeting HSC70 (300 nM), the HSC70 level was significantly reduced in GFP-positive neurons, but the expression of GAPDH was not changed (Fig. 4A). In the HSC70-knocked down neurons, density of pNF-H-positive axon was significantly greater than the density in the control siRNA-transfected neurons (Fig. 4B), suggesting that the reduction in the HSC70 level has a direct effect on axonal growth activity.

### α-Tubulin was identified as a binding partner (client protein) of HSC70

Next, we examined the downstream signaling of HSC70, which directly relates to axonal regrowth and memory function. To work as a molecular chaperone, HSC70 binds to several client proteins and promotes their degradation and folding; therefore, we hypothesized that the chaperone function of HSC70 is increased by Aβ deposition. Therefore, we explored the binding partners of HSC70, the expression of which was increased by Aβ treatment.

**Figure 5:**
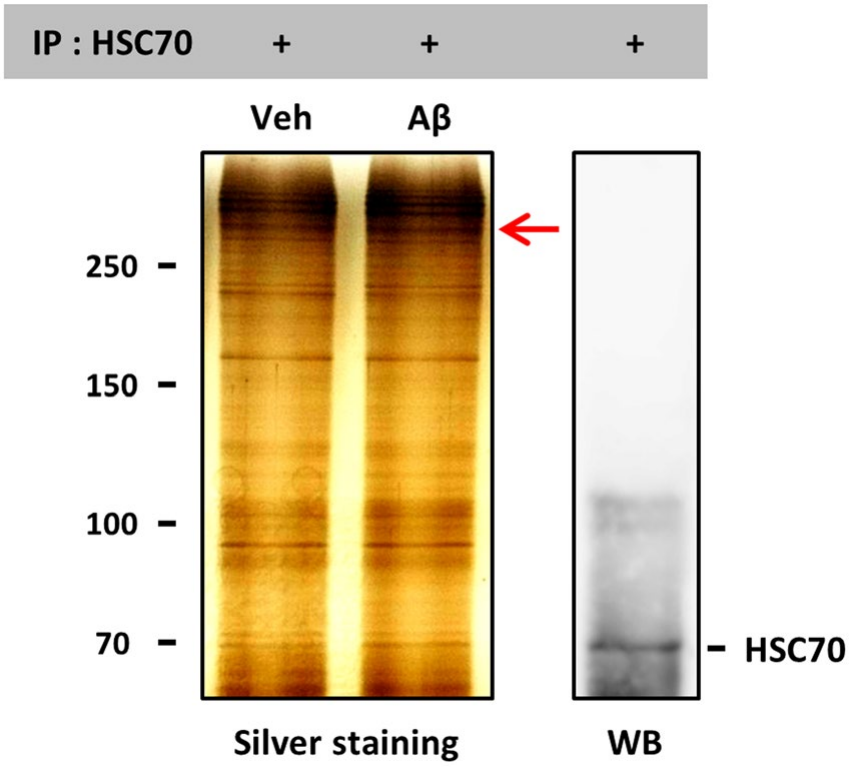
α-Tubulin was identified as a binding (client) protein of HSC70. Mouse cortical neurons were cultured for three days and then treated with vehicle solution or Aβ_25–35_ (10 µM) for 30 min at 37 °C. Cell lysates were prepared and co-immunoprecipitated with the anti-HSC70 antibody. Precipitated proteins were mildly heated at 65 °C to maintain the complex formation of HSC70 and its binding partners, and electrophoresed to perform silver staining or WB for HSC70. The protein band in which the intensity was increased by Aβ treatment (red arrow) was cut out and prepared for MS analysis. The images in the figure were cropped and the full-length gels are presented in Supplementary Fig. 3.

Three days after culture, neurons were treated with or without Aβ (10 µM) for 30 min. After co-immunoprecipitation with the anti-HSC70 antibody, precipitated proteins were mildly heated at 65 °C to maintain the complex formation of HSC70 and its binding partners, and electrophoresed to perform silver staining or WB. Immunoprecipitation of HSC70 was confirmed by WB. Among the proteins that co-precipitated with HSC70, the protein that was densely stained on a silver stained gel in the Aβ-treated lane (red arrow; the quantitative intensity was 122% compared with vehicle-treated lane) was cut out. The protein band was excised, digested with trypsin (Promega, Madison, WI, USA), and identified by nano LC-MS/MS (Japan Bio Services, Saitama, Japan) with searching in MASCOT database (Fig. 5). The band was determined to include α-tubulin (protein sequence coverage: 6%, score: 44), suggesting that α-tubulin is a candidate client protein of HSC70, especially in the case of Aβ stimulation. The full-length gels are presented in Supplementary Fig. 3.

The tubulin dimer of α-tubulin and β-tubulin was already known to associate with HSC70^14^. However, the subsequential signaling of their association in neurons had never been clarified. We focused on α-tubulin as a HSC70-associated molecule, and investigated the function of α-tubulin in Aβ-treated and diosgenin-treated neurons.

### Diosgenin prevented the α-tubulin decline and normalized the axonal disruption caused by Aβ treatment

**Figure 6:**
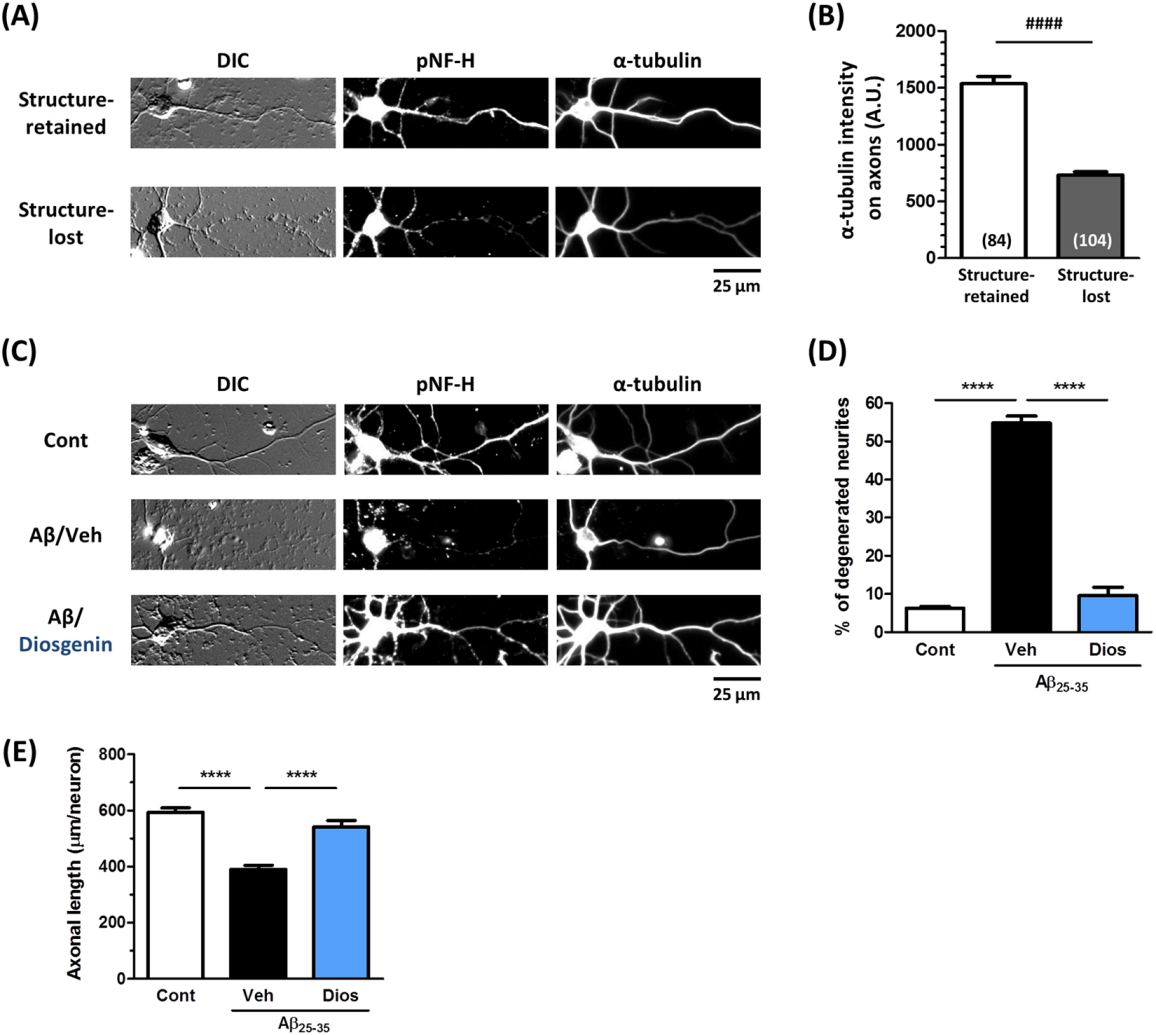
Relationship between α-tubulin expression and axonal formation in Aβ- and diosgenin-treated neurons. (**A**,**B**) Mouse cortical neurons were cultured for two days and treated with Aβ_25–35_ (10 µM) for one day. After the medium was removed, the neurons were treated with fresh medium for four days. Neurons were fixed and double-immunostained for α-tubulin and pNF-H or observed with DIC. (**A**) Structure-retained axons and dot-like or swollen structure-lost axons were observed. (**B**) α-Tubulin expression was quantified in the structure-retained or structure-lost axons. ^####^p < 0.0001, two-tailed unpaired *t*-test. n = 84–104 axons were quantified for the analysis. **(C**–**E)** Mouse cortical neurons were cultured for two days and treated with Aβ_25–35_ (10 µM) for one day, followed by treatment with diosgenin (1 µM) or vehicle solution (0.1% EtOH) for four days. (**C**) Neurons were fixed and double-immunostained for α-tubulin and pNF-H or observed with DIC. (**D**) The percentage of structure-lost (low expression of α-tubulin) axons is shown for each treatment group. (**E**) Densities of pNF-H-positive axons were quantified for each treatment. ****p < 0.0001, two-tailed one-way ANOVA *post hoc* Bonferroni’s multiple comparison test. (**D**) n = 3 photos, (**E**) n = 12–25 photos were quantified for these analyses.

Next, we investigated the relationship between α-tubulin and axonal degeneration. Two days after culture, neurons were treated with Aβ_25–35_ for one day. After the medium was removed, the neurons were treated with fresh medium for another four days, and DIC (differential interference contrast) images as well as fluorecent images showing pNF-H-positive axons and α-tubulin-positive structures were observed (Fig. 6A).

In the DIC images, dot-like or swollen degenerated axonal traces (Structure-lost) as well as definite axonal structures (Structure-retained) were observed. The definite neurites were pNF-H-positive and expressed high level of α-tubulin. However, in the dot-like neurite, only the basal part adjacent to the cell body was slightly pNF-H-positive, but the distal portion was pNF-H-negative and only exhibited faint α-tubulin staining. In the Aβ_25–35_-treated neurons, we could distinguish pNF-H-positive structure-retained axons and pNF-H-negative structure-lost axons, and the expression level of α-tubulin in the structure-lost degenerated axons was decreased to 46% of the level in the structure-retained axons (Fig. 6B).

Next, we evaluated the effect of diosgenin on the ratio of structure-retained and structure-lost axons under Aβ_25–35_ treatment based on the DIC and pNF-H-stained images. Two days after culture, neurons were treated with or without Aβ_25–35_ for one day, followed by four days of treatment of diosgenin or vehicle solution. Control neurons (no Aβ_25–35_ treatment) showed a very low ratio (6%) of structure-lost axons. Aβ_25–35_ treatment elevated the ratio of structure-lost axons to 55%, but diosgenin treatment decreased the ratio to 10% (Fig. 6C,D, respectively). On the other hand, control peptide Aβ_35–25_ showed a very low ratio of structure-lost axons as same levels as control neurons (Supplementary Fig. 4). The axonal densities were also quantified in the same conditions by measuring the length of the pNF-H-positive axons. While the axonal length was significantly decreased by Aβ_25–35_ treatment, post treatment with diosgenin increased it (Fig. 6E). In addition, the number and length of the structure-retained axons were reduced by Aβ_25–35_ treatment but normalized by diosgenin.

## Discussion

Our previous study found that diosgenin significantly restored the Aβ-induced axonal atrophy and memory deficits in 5XFAD mice^2^. This study investigated the key signaling of diosgenin.

By using 2D-PAGE analysis, we comprehensively explored proteins whose expression level was changed in the 5XFAD mouse cortex, and analyzed some of them by MALDI-TOF/MS. HSC70 was identified as a protein whose expression was increased in the 5XFAD mouse and drastically decreased by diosgenin administration. Other than HSC70, three candidate proteins (A-X actin, γ-actin, and v-type proton ATPase catalytic subunit A) also showed increase in 5XFAD mice and decrease by diosgenin administration. The function of actin family has already been well studied to associate with AD brains^15^. On the other hand, there is no report about the expression level of v-type proton ATPase catalytic subunit A in AD patient brains. The expression level of HSC70 is higher in AD model mice and AD patient brains than in wild-type mice and healthy controls, respectively^10^. We focused on HSC70 due to its relevance with AD. Immunocytochemistry and WB analysis confirmed that the expression level of HSC70 was truly increased by Aβ treatment in neurons and higher in the 5XFAD mouse cortex than in the wild-type cortex; diosgenin treatment then decreased its expression to the same levels as the normal condition. Diosgenin-induced axonal regrowth and memory enhancement is mediated by 1,25D_3_-MARRS signaling. As in our previous studies^2^, the present study showed that the diosgenin-induced reduction in HSC70 was also mediated by 1,25D_3_-MARRS (Fig. 3), indicating that the change of HSC70 expression is certainly included in diosgenin signaling.

HSC70 preserves the accumulation of phosphorylated tau in AD pathology^16^, and the neurofilament medium chain (NF-M) interacts with HSC70 and is degraded by the ubiquitin proteasome system (UPS)^17^. However, the direct relationship between HSC70 expression and axonal growth has not been clarified. Figure 4 shows that knockdown of HSC70 promoted axonal growth in neurons, the first finding indicating that the reduction in HSC70 may be critical for axonal growth. As a molecular chaperone, HSC70 has a variety of client proteins. We were very interested in determining which molecule might be recruited to HSC70 under conditions of Aβ stimulation. As shown in Fig. 5, α-tubulin was determined to be one of the partners of HSC70 in neurons. Analyzed the band with more than 250 kDa molecule weight in Fig. 5, might be a complex of HSC70, α-tubulin and others. Since the tubulin dimer of α-tubulin and β-tubulin is already known to associate with HSC70^14^, our result, the association of HSC70 and α-tubulin was unsurprising. Therefore, we investigated the relationship between axonal formation and α-tubulin expression. In Fig. 6A,B, structure-retained (healthy) axons were distinguished from structure-lost (degenerated) axons, which was exhibited low expression of α-tubulin in Aβ-treated neurons. The ratio of low α-tubulin axons was drastically decreased by diosgenin treatment (Fig. 6C,D, respectively). As shown in Fig. 2, diosgenin decreased the HSC70 levels in neurons. Taken together, we hypothesized that the diosgenin-triggered HSC70 decline leads to normalization of α-tubulin expression under Aβ treatment. HSC70 is well known to degrade its client proteins by UPS^18^ or chaperone-mediated autophagy (CMA)^19^. CMA requires a specific motif, the KFERQ motif, which is lacking in α-tubulin. Future studies should aim to investigate HSC70-mediated α-tubulin degradation and its mechanism.

α-Tubulin is one of the main components of microtubules, polymerization of which is necessary to promote axonal growth^20^. Sadleir’s group and others have thoroughly investigated the role of tubulin in AD pathologies. Aβ disrupts the organization of tubulin-positive microtubules in neurons. Tubulin isoforms are abnormally accumulated in beta-site amyloid precursor protein-cleaving enzyme (BACE) 1-positive peri-plaque dystrophic neurites in 5XFAD mouse brains^21^. Furthermore, the expression levels of α-tubulin and tubulin-positive axonal processes are lower in AD patient brains than in controls^22^. Based on these observations, the reduction in α-tubulin is probably related to neurite disruption in AD. However, the cellular and molecular mechanisms leading to the decline in α-tubulin in AD had never been clarified. To our knowledge, our findings are the first to suggest that HSC70 may control the decline in α-tubulin. Moreover, diosgenin has been identified as a compound that can induce a reduction in HSC70 expression in AD pathology.

Our recent studies have demonstrated that a functional inhibitor of HSC70, VER-155008, restores Aβ-induced axonal atrophy in neurons, and recovers object recognition, location, and episodic-like memories in 5XFAD mice^23^. In 5XFAD mice brain, abnormal swollen axons are reduced by VER-155008 administration. During the administration, abnormal behaviors are not observed in mice. Furthermore, VER-155008 transfers into the brain after its administration, suggesting that inhibiting HSC70 function in the brain is truly effective and safe in AD.

This study showed that HSC70 expression is a key regulator of axonal formation in AD pathologies. Moreover, our data indicate that HSC70 expression is controllable by diosgenin. Therefore, this study suggests that downregulating HSC70 expression can be a new therapeutic target for AD treatment.

## Methods

All experiments were performed in accordance with the Guidelines for the Care and Use of Laboratory Animals of the University of Toyama. The committee for Animal Care and Use at the Sugitani Campus of the University of Toyama approved the study protocols. The approval number for the animal experiments is A2017INM-1. All efforts were made to minimize the number of animals used.

### Animal studies

Transgenic mice (5XFAD) were obtained from the Jackson Laboratory (Bar Harbor, ME, USA) and maintained by crossing hemizygous transgenic mice with B6/SJL F1 breeders. To investigate the effect of diosgenin on 5XFAD mice, we used hemizygous transgenic 5XFAD mice (males, 5–6 months old, Fig. 1; females, 7–8 months old, Fig. 2C) and non-transgenic wild-type littermate mice (male, 5–6 months old, Fig. 1; females, 7–8 months old, Fig. 2C). All mice were housed with free access to food and water and kept in a controlled environment (22 ± 2 °C, 12-h light/dark cycle starting at 7:00 am)

### Novel object recognition test

Diosgenin (Tokyo Chemical Industry, Tokyo, Japan) dissolved in sesame oil (Kaneda, Tokyo, Japan) or vehicle solution (sesame oil) was orally administered once a day for 15 days (Fig. 1) or 18 days (Supplementary Fig. 2). On the last day (Fig. 1) or day 14 (Supplementary Fig. 2) of administration, a novel object recognition test was performed as described previously^24^. Testing was carried out in a dimly illuminated room.

### Proteins identification using mass spectrometry analysis

The mouse brain cortex was homogenized with M-PER (Thermo Scientific, Rockford, IL, USA) containing 1× Halt protease & phosphatase inhibitor cocktail (Thermo Scientific). The brain lysate (600 µg per each) was loaded on 2D-PAGE (GENOMINE, Inc., Korea), and the separated proteins were stained using colloidal Coomassie Brilliant Blue (CBB). Proteins whose expression level were changed in the 5XFAD mouse cortex, but the change was drastically diminished by diosgenin administration were chosen. The focused proteins were cut out and subjected to matrix-assisted laser desorption/ionization-time-of-flight mass spectrometry (MALDI-TOF MS) analysis (MATRIX SCIENCE).

### Primary culture and immunocytochemistry

Embryos were removed from a pregnant ddY mouse (Japan SLC, Shizuoka, Japan) at 14 days of gestation as described previously^2^. The Aβ_35–35_ and Aβ_25–35_ was previously incubated for four days at 37 °C for aggregation. Cells were cultured for three days and treated with 10 μM Aβ_35–35_ or 10 μM Aβ_25–35_ (Sigma-Aldrich, St. Louis, MO, USA) for three days and then 0.1 or 1 µM diosgenin or vehicle solution (ethanol) for four days (Fig. 2A,B). Three days after Aβ_25–35_ treatment, cells were incubated with a neutralizing antibody, followed by a 15-min incubation with the control (normal rabbit IgG, 1:1000, Santa Cruz, CA, USA) or anti-rabbit 1,25 D_3_-MARRS antibody (Ab099 clone, 1:1000, gifted by Dr. Ilka Nemere) Then, diosgenin (0.1 or 1 µM) or vehicle solution (0.1% ethanol) was added to the cells for four days (Fig. 3). Cells were cultured for two days and treated with 10 μM Aβ_25–35_ for one day and then 1 µM diosgenin or vehicle solution (0.1% ethanol) for four days (Fig. 6). The cells were fixed with 4% paraformaldehyde and immunostained at 4 °C for 24 h with an antibody against the phosphorylated axonal neurofilament H subunit (anti-mouse pNF-H antibody; monoclonal IgG_1_, 1:250, Covance, Princeton, NJ, USA) as an axonal marker, the anti-mouse HSC70 antibody (monoclonal IgM, 1:300, Abcam, Cambridge, United Kingdom), the anti-mouse GAPDH antibody (monoclonal IgG_1_, 1:100, Applied Biological Materials Inc., Canada), or anti-rabbit α-tubulin antibody (polyclonal IgG, 1:200, Abcam). Alexa Fluor 488- or 568-conjugated goat anti-mouse IgG (1:400), Alexa Fluor 350-conjugated goat anti-mouse IgM (1:400), Alexa Fluor 594-conjugated goat anti-mouse IgG_1_ (1:400), or Alexa Fluor 488-conjugated goat anti-rabbit IgG (1:400) were used as secondary antibodies. Fluorescence images (864.98 µm × 645.62 µm) were captured using a fluorescence microscopy system (Carl Zeiss, Oberkochen, Germany). The lengths of the pNF-H-positive axons and the expression of HSC70 or GAPDH in neurons were measured using MetaMorph version 7.8 (Molecular Devices, Sunnyvale, CA, USA). The percent (%) of healthy or degenerated neurites and the α-tubulin intensity on neurites were quantified by the image analyzing software, ImageJ.

### siRNA transfection

siRNAs were transfected into mouse cortical neurons (ddY E14) according to the manufacturer’s protocol for nucleofection (Lonza, Basel, Switzerland). Mouse cortical neurons (5.0 × 10^6^ cells) were mixed with 300 nM siHSC70 (a mixture of two sequences of Stealth siRNA for HSC70, Life Technologies) or 300 nM control siRNA (Select Negative Control siRNA #1, Thermo Scientific) and 2 µg GFP vector, and electroporated with an Amaxa Nucleofector (Lonza). Four days after the transfection, cells were fixed and immunostained with the anti-HSC70 antibody (1:300), anti-GAPDH antibody (1:100), and anti-GFP antibody (monoclonal IgG_2a_, 1:500, nacalai tesque, Kyoto, Japan), or the anti-pNF-H antibody (1:250) and anti-GFP antibody. The expression of HSC70 and density of pNF-H-positive axons in GFP-positive neurons were measured using ImageJ. The appropriate concentration of siRNA and the appropriate duration for knockdown were determined previously.

### Western blot

Mouse brain cortex was homogenized with M-PER (Thermo Scientific) containing 1× Halt protease & phosphatase inhibitor cocktail (Thermo Scientific). The brain lysate (3 µg/lane) was loaded on SDS-PAGE for western blot (WB) analysis. After blocking the membrane with 5% skim milk (Wako Pure Chemical Industries, Japan) in 0.1% Tween-TBS for 30 min at room temperature, the anti-mouse HSC70 antibody (1:4000, Abcam) and anti-rabbit β-actin antibody (1:1000, Cell Signaling Technology) were used as the primary antibodies, and HRP-conjugated goat anti-mouse IgM (1:2000, Santa Cruz) and HRP-conjugated goat anti-rabbit IgG (1:2000, Santa Cruz) were used as the secondary antibodies. Amersham™ ECL™ Western Blotting Detection Reagents (Sigma-Aldrich) was used for the detection of the bands according to the manufacture’s protocol.

### Immunoprecipitation and SDS-PAGE

Mouse primary cortical neurons (ddY E14) were cultured for three days followed by a 30-min incubation with Aβ_25–35_ or vehicle solution (dH_2_O). After the cells were washed with PBS, they were lysed with M-PER solution containing 1× Halt protease & phosphatase inhibitor cocktail. To avoid detection of the proteins which directly bind with Dynabeads™ Protein G (Life Technologies) on the silver staining, cell lysates was previously incubated with Dynabeads™ Protein G for 10 min at room temperature, and the non-specific binding proteins were removed from the cell lysates. The cell lysates (50 µg) were then incubated with the anti-rabbit HSC70 antibody (4.27 µg; monoclonal, Abcam) for 30 min at 4 °C. Finally, fresh Dynabeads™ Protein G was incubated for 20 min at 4 °C, and the samples were mildly heated at 65 °C for 5 min. After immunoprecipitation, the proteins were loaded on SDS-PAGE for silver staining and WB. For sliver staining, SilverQuest™ Silver Staining Kit (Thermo Scientific) was used according to the manufacture’s protocol. For WB, the anti-rabbit HSC70 antibody (1:3000, Abcam) was used as primary antibody, and HRP-conjugated goat anti-rabbit IgG (1:2000, Santa Cruz) was used as secondary antibody.

### Statistical analysis

Statistical comparisons were performed using one-way analysis of variance (ANOVA) with *post hoc* Dunnett’s tests, Student’s unpaired *t*-tests and one sample *t*-tests in GraphPad Prism 5 (GraphPad Software, La Jolla, CA, USA). Values of p < 0.05 were considered significant. The data are presented as the mean ± SEM.

## Electronic supplementary material

Supplementary Information
