# Supplementary material for: Diosgenin restores Aβ-induced axonal degeneration by reducing the expression of heat shock cognate 70 (HSC70)
Source: Sci Rep. 2018 Aug 3;8:11707. doi: 10.1038/s41598-018-30102-8 (PMC6076317; doi:10.1038/s41598-018-30102-8)
Supplement: Supplementary file 1 — Supplementary Information [file 41598_2018_30102_MOESM1_ESM.pdf]

Supplementary Information

Diosgenin restores Aβ-induced axonal degeneration by reducing the expression of heat shock cognate 70 (HSC70)

Ximeng Yang[1], Chihiro Tohda*[1]

[1]Division of Neuromedical Science, Department of Bioscience, Institute of Natural Medicine, University of Toyama, Toyama, Japan

*To whom correspondence:
Chihiro Tohda, Ph.D.
Division of Neuromedical Science, Institute of Natural Medicine, University of Toyama, 2630 Sugitani, Toyama 930-0194, Japan
Phone (FAX): +81-76-434-7646
E-mail: chihiro@inm.u-toyama.ac.jp

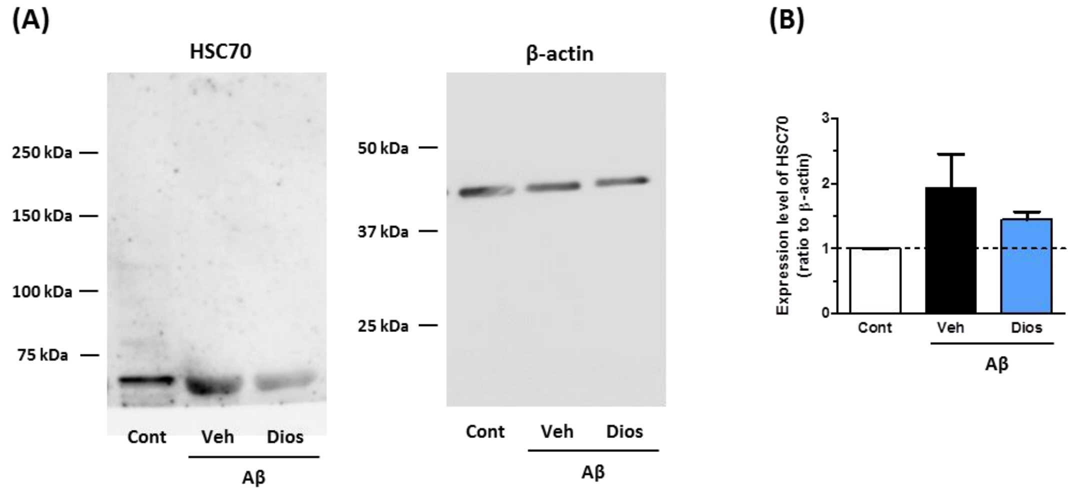

Supplementary Figure 1. Diosgenin reduced the expression level of HSC70 in neuron lysates. Mouse cortical neurons (ddY, E14) were cultured for three days and then treated with or without A$\beta_{25\text{-}35}$ (10 µM) for three days. After the three days of A$\beta_{25\text{-}35}$ treatment, neurons were treated with diosgenin (1 µM) or vehicle solution (0.1% EtOH) for four days. After the cells were washed with PBS, they were lysed and loaded on SDS-PAGE for WB analysis. **(A)** Proteins were detected by anti-HSC70 antibody or β-actin antibody, respectively. A whole membrane was cropped around 60 kDa into two pieces, upper one (for anti-HSC70) and lower one (for anti-β-actin) before WB. **(B)** Quantitative value for the expression levels of HSC70 (ratio to β-actin), n = 3 samples (2–4 lanes per a sample were quantified for the analysis).

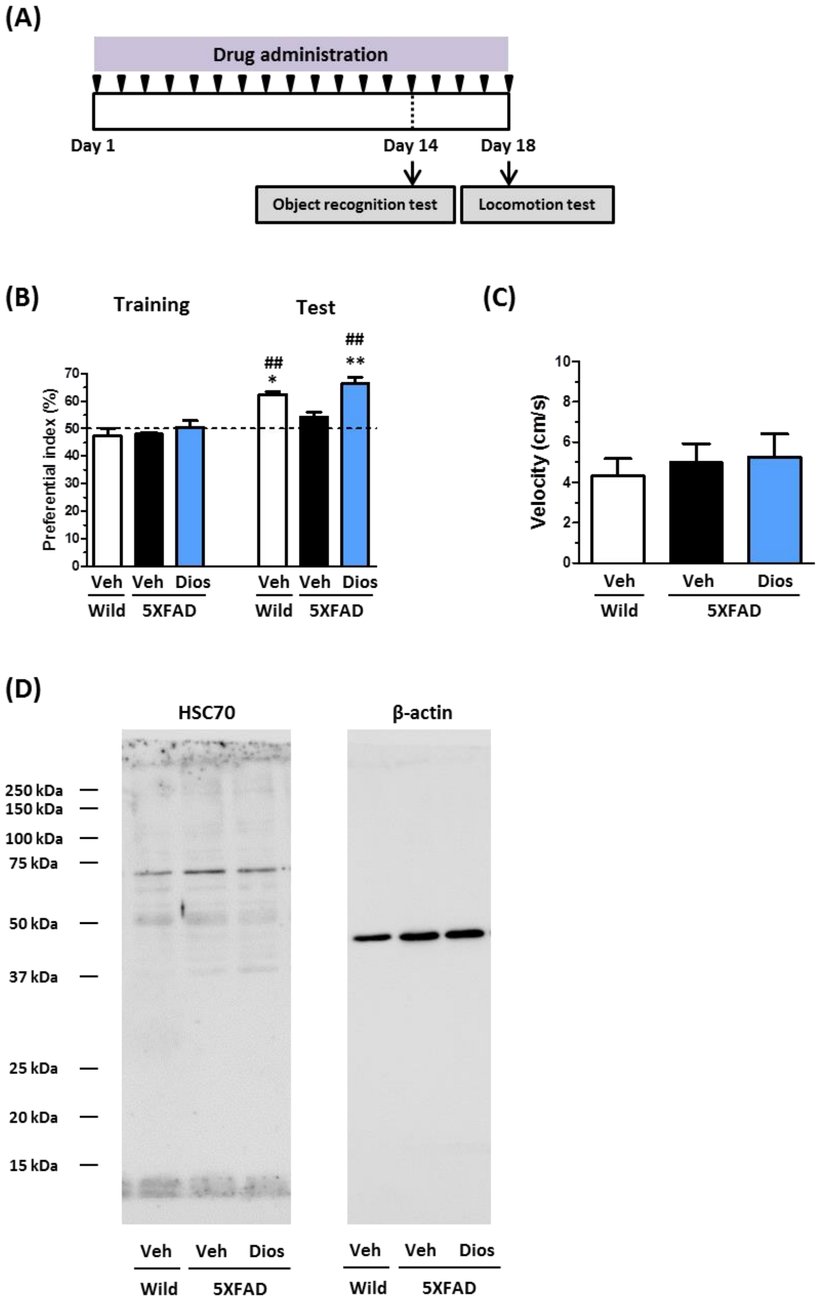

Supplementary Figure 2. Diosgenin administration recovered memory deficits in the 5XFAD mice. Wild-type and 5XFAD mice (Female, 7–8 months old) were treated with diosgenin (0.1 μmol/kg/day, p.o.) or vehicle solution (sesame oil) for 18 days. **(A)** The time schedule of this study. On administration day 14, an object recognition memory test was performed. On the last day of administration, locomotion test was performed. **(B)** The preferential indexes of the training and test session are shown. *$p < 0.05$, **$p < 0.01$ vs 5XFAD/Veh, one-way ANOVA *post hoc* Dunnett's test, ##$p < 0.01$ vs 50%, one sample *t*-test, n = 4 mice. **(C)** The velocity (cm/s) within 10 min is shown. One-way ANOVA *post hoc* Dunnett's test, n = 4 mice. **(D)** The full-length WB images for the Figure 2C in the main text.

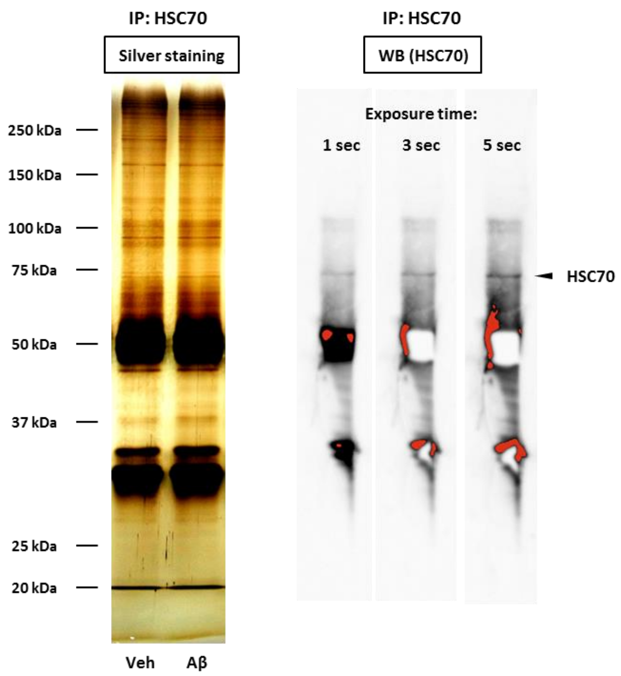

Supplementary Figure 3. The full-lengths gels for the Figure 5 in the main text.

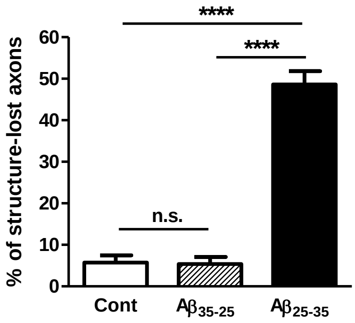

Supplementary Figure 4. The effects of Aβ$_{35\text{-}25}$ (a negative control peptide) and Aβ$_{25\text{-}35}$ on axonal structures. Mouse cortical neurons were cultured for two days and treated with Aβ$_{35\text{-}25}$ (10 µM) or Aβ$_{25\text{-}35}$ (10 µM) for one day. After the medium was removed, the neurons were treated with fresh medium for four days. Then, neurons were fixed and double-immunostained for α-tubulin and pNF-H or observed with DIC. The percentage of structure-lost (low expression of α-tubulin) axons were quantified for each treatment. ****$p < 0.0001$, two-tailed one-way ANOVA *post hoc* Bonferroni's multiple comparison test. n = 3 photos were quantified for the analysis.
